# Awareness of the implementation of stable iodine prophylaxis by parental guardians living in the urgent protective action planning zone of an operating nuclear power plant in Japan

**DOI:** 10.1186/s12889-021-12349-5

**Published:** 2021-12-08

**Authors:** Hitomi Matsunaga, Makiko Orita, Yasuyuki Taira, Noboru Takamura

**Affiliations:** Department of Global Health, Medicine and Welfare, Atomic Bomb Disease Institute, Nagasaki University, 1-12-4 Sakamoto, Nagasaki, 852-8523 Japan

**Keywords:** Stable iodine, Pre-distribution, Urgent protective action planning zone, Child

## Abstract

**Purpose:**

The aim of this study was to clarify the characteristics and awareness of the need for protection against ionizing radiation, such as sheltering, evacuation, and implementing stable iodine prophylaxis, of guardians parenting young children living in an urgent protective action planning zone (UPZ) of an operating nuclear power plant in Japan.

**Methods:**

Self-administered questionnaires were distributed to approximately 3000 guardians through 26 kindergartens located within a UPZ. Responses were obtained from 1172 who lived in the UPZ and were included in the analysis.

**Results:**

Of the 1172 guardians, 460 (39.2%) responded that sheltering is not useful to reduce the dose of radiation exposure. On the other hand, 395 (33.7%) guardians responded that implementing stable iodine (SI) prophylaxis could prevent exposure from all radionuclides, and 876 (74.7%) responded that pregnant women should also implement SI prophylaxis in a nuclear emergency. Furthermore, 83.0% (973) responded that they wanted to receive pre-distribution of stable iodine (PDSI) for their children. On the other hand, 38.9% (456) of guardians had not known about SI before the study, and 71.8% (841) of guardians felt anxious about implementing SI prophylaxis for their children.

**Conclusion:**

Most guardians had expectations regarding SI and received PDSI, but they felt anxious about implementing SI prophylaxis for their children. It is essential that guardians living in the UPZ of restarted nuclear power plants be educated, and that risk communication about protection against ionizing radiation, including the side effects of implementing SI prophylaxis and radiation health effects, be conducted.

## Background

Following the Fukushima Daiichi Nuclear Power Plant (FDNPP) accident in 2011 [1–4], the NRA established the “Nuclear Emergency Response Guidelines” to provide an appropriate and smooth nuclear emergency response for the vast numbers of residents living around a nuclear power plant (NPP) [5]. The NRA defined protection against ionizing radiation based on distance from an NPP such as the precautionary action zone (PAZ) and the urgent protective action planning zone (UPZ). The PAZ is an area within a radius of 5 km from the NPP in which evacuation is conducted proactively before the release of radioactive materials if an accident occurs at an NPP. On the other hand, the UPZ is an area within a radius of 30 km outside the PAZ, in which the priority is to shelter in their house or a public shelter if an accident occurs at an NPP, then according to the nuclear accident severity stage or radiation air dose rate from the release of radionuclides, it is recommended that people begin evacuation from their residential area and start to receive support from the municipality or ministries of Japan for protection against ionizing radiation in the UPZ [6, 7]. Therefore, the residents living in a UPZ must deepen their understanding of protection against ionizing radiation when an NPP accident occurs.

Following the FDNPP accident, a significant increase in thyroid cancer has not been recorded [1, 2], although thyroid cancer became a serious issue for the generation of children at the time of the Chernobyl nuclear power plant accident in 1986 [8, 9]. The characteristics of thyroid cancer caused by internal radiation exposure showed it developed more often among younger people [10, 11]. From these lessons, implementation of stable iodine (SI) prophylaxis in a nuclear accident has been introduced in Japan, as well as in other countries [12]. Among the various actions to protect against ionizing radiation, implementing SI prophylaxis is a key strategy for preventing internal exposure from radioactive iodine [13]. In Japan, pre-distribution of stable iodine (PDSI) was introduced to residents under 40 years of age living only in a PAZ since 2014, so that they could take it immediately and evacuate when a nuclear accident occurs [14]. However, since 2019, PDSI for UPZ residents has been started by five of 21 administrations of the UPZ regions throughout Japan [6].

**Fig. 1:**
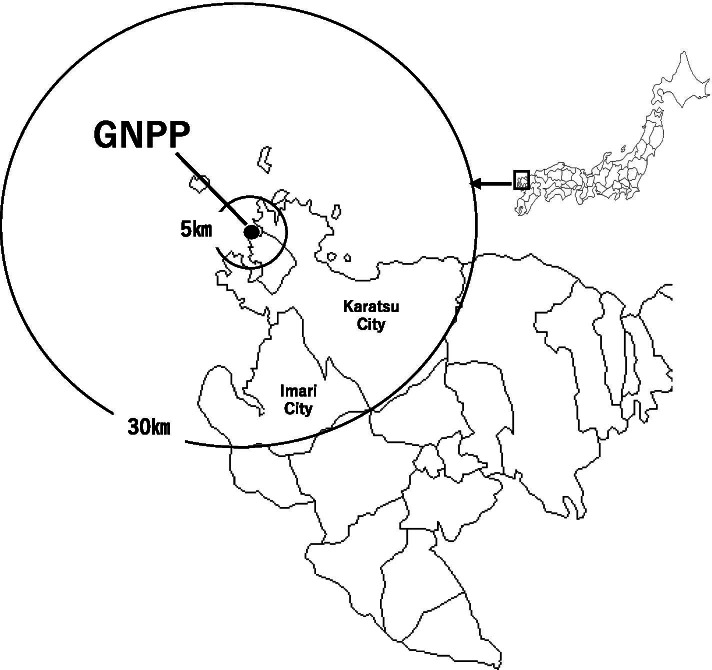
Location of the Genkai Nuclear Power Plant (GNPP) in Saga Prefecture, Japan

Kyushu Electric Power Co., Inc.’s Genkai nuclear power plant (GNPP) is located in Genkai Town, Saga Prefecture, Japan (Fig. 1). The UPZ of the GNPP includes Karatsu City (population of 121,148 people and 48,638 households) and Imari City (population of 56,063 people and 22,911 households) [15]. The GNPP has four reactors, two of which have been restarted since 2018 [16], and PDSI was introduced for the PAZ residents. In addition, PDSI was also started for UPZ residents, who only applied for it since 2019. However, there were few PDSI applicants among the UPZ residents, especially the younger generation, in the GNPP area [17]. In our previous study of the PAZ of GNPP, we found that knowing that children are a priority for implementing SI prophylaxis and knowing about the booklet regarding SI published by the local government were independently associated with PDSI for children. In contrast, we asked the guardians living in the PAZ of the GNPP who did not receive PDSI about their reasons, and they responded that they had anxiety about the side effects of SI, distrusted the effectiveness of SI, and thought that the procedures for receiving SI were complicated [18]. The local government of the area in which an NPP is located has the responsibility for planning protection against ionizing radiation, holding nuclear energy disaster prevention drills, or annual meetings for local residents. Furthermore, local governments also have the responsibility for providing PDSI and instructing residents in the UPZ about how to implement SI prophylaxis and storage at home, and about the timing of implementing SI prophylaxis during a nuclear accident [6].

However, the awareness of the need for protection against ionizing radiation, including sheltering, SI, PDSI, and implementing SI prophylaxis, of the residents parenting young children living in areas where an NPP was operating has not been examined. Therefore, the aim of the present study was to clarify the characteristics and awareness of the need for protection against ionizing radiation, such as sheltering, evacuation, SI, PDSI, and implementing SI prophylaxis, of guardians parenting young children living in UPZ of an operating NPP in Japan. The study results contribute to not only making a plan for protection against ionizing radiation in the local municipalities based on the awareness of the residents, but also protection against ionizing radiation for guardians parenting young children at high risk of internal thyroid exposure.

## Methods

### Study participants

This study was conducted from January to June 2020 in two municipalities (Karatsu City and Imari City) located within 30 km (UPZ) of the GNPP in Saga Prefecture, Japan (Fig. 1). Potential participants were informed about the ethical aspects of the study and return of the questionnaire was considered to indicate their informed consent. A self-administered questionnaire was distributed to approximately 3000 guardians with children through a random selection of 26 of approximately 40 kindergartens in both cities located only within the UPZ, and responses were obtained from 1863. After excluding incomplete responses, complete responses were obtained from 1785 guardians (valid response rate 95.8%), and data were obtained from 20 (1.1%) guardians who lived within 5 km of the GNPP [PAZ], 1172 (65.7%) who lived within 30 km of the GNPP [UPZ], 229 (12.8%) who lived more than 30 km from the GNPP, and 364 (20.4%) whose distance from the GNPP was unknown. The aim of the study was to clarify the characteristics and awareness of the need for protection against ionizing radiation of the guardians living in the UPZ of an operating NPP. Therefore, in the study, the data of 1172 guardians were included in the analysis.

### Data collection

In the self-administered questionnaire, a brief explanation regarding the giving of SI to their children was provided to those who were not familiar with it, as follows. “Stable iodine tablets prevent thyroid exposure from a substance called radioactive iodine for only 24 hours. It is stockpiled in municipal government buildings so that it can be distributed immediately in the event of a nuclear accident. There is a sweet jelly that can be taken to protect an unborn baby even during pregnancy, and it is easy to take even for newborns.” The guardians were asked whether they had known about SI before the study, whether they wanted to receive PDSI for their children, and whether they wanted to implement SI prophylaxis for their children in a nuclear emergency in “yes/no” questions. Furthermore, “yes/no” questions regarding whether they had participated in a nuclear emergency drill and whether they had known of a public shelter to evacuate to during a nuclear emergency were also asked.

Regarding protection against ionizing radiation, whether they thought that sheltering is useful to reduce the dose of radiation exposure, whether they thought that SI could prevent exposure from all radionuclides, whether they thought that pregnant women should also implement SI prophylaxis in a nuclear emergency, and whether they felt anxious about implementing SI prophylaxis for their children were also asked. In addition, whether they wanted to participate in a nuclear emergency drill and a lecture about the health effects of radiation exposure was also asked. For these questions, respondents were asked to choose one of the following four responses: “yes”, “probably”, “probably no”, or “no”. The “yes” and “probably” responses were classified as “Yes”, and the “probably no” and “no” responses were classified as “No”. Demographic factors, including sex, age (< 30, 30–39, 40–49, and ≥ 50 years), number of children under 18 years of age, social factors, and distance from the GNPP to their house (PAZ, UPZ, > 30 km, and unknown), were also included in the questionnaire. In this study, SI refers to the name of the drug, PDSI refers to the act of pre-distributing SI, and implementing SI prophylaxis refers to the administration of a drug (SI).

### Statistical analysis

After the descriptive statistics were calculated, those who did and did not want to receive PDSI were defined as “Group 1” and “Group 2”, respectively. Differences between Group 1 and Group 2 were evaluated using chi-squared tests. Then, factors independently associated with those who wanted to receive PDSI (Group 1) were determined using binary logistic regression analysis. Before performing the binary logistic regression analysis, it was confirmed that, for each variable with a *p*-value < 0.01, the correlation coefficient (r) in the chi-squared tests was < 0.8, to exclude the possibly of collinearity. The variables included in the binary logistic regression analysis were: “thought that pregnant woman should also implement SI prophylaxis in a nuclear emergency”; “want to participate in a lecture about the health effects of radiation exposure”; “thought that implementing SI prophylaxis can prevent exposure from all radionuclides”; “implementing SI prophylaxis can prevent exposure from all radionuclides”; “did know about SI before the study”; and had anxiety about implementing SI prophylaxis for their children. The statistical analysis was performed using IBM SPSS Statistics version 19, SPSS Japan, Tokyo, Japan.

## Results

Table 1 shows the characteristics of the study participants. Of the 1172 guardians, 1011 (86.3%) were female, 121 (10.3%) were < 30 years, 729 (62.2%) were 30–39 years, 315 (26.9%) were 40–49 years, and 7 (0.6%) were ≥ 50 years old. Regarding the number of children, 227 (19.4%) had one child, 493 (42.1%) had two children, and 452 (38.5%) had three or more children. Furthermore, 957 (81.7%) had not participated in a nuclear emergency drill, 748 (63.9%) wanted to participate in a nuclear emergency drill, and 676 (57.7%) wanted to participate in a lecture about the health effects of radiation exposure in the guardians. Of the total guardians, 712 (60.8%) thought that sheltering is useful to reduce the dose of radiation exposure, and 487 (41.6%) did not know of a public shelter to evacuate to during a nuclear emergency. Of the total guardians, 716 (61.6%) responded that they knew about SI before the study, 395 (33.7%) thought that implementing SI prophylaxis can prevent exposure from all radionuclides, 992 (84.6%) wanted to implement SI prophylaxis for their children in a nuclear emergency, and 876 (74.7%) responded that pregnant women should also implement SI prophylaxis in a nuclear emergency. On the other hand, 841 (71.8%) responded that they felt anxious about implementing SI prophylaxis for their children.

**Table 1:** Characteristics of the guardians in the UPZ. *N* = 1172

	n	%
Sex		
Male	161	13.7
Female	1011	86.3
Age		
< 30 years	121	10.3
30–39 years	729	62.2
40–49 years	315	26.9
≥ 50 years	7	0.6
How many children under 18 years of age?		
1	227	19.4
2	493	42.1
3 or more	452	38.5
Have you participated in a nuclear emergency drill?		
Yes	215	18.3
No	957	81.7
Do you want to participate in a nuclear emergency drill?		
Yes	119	10.2
Probably	629	53.7
Probably No	372	31.7
No	52	4.4
Do you want to participate in a lecture about the health effects of radiation exposure?		
Yes	89	7.6
Probably	587	50.1
Probably No	423	36.1
No	73	6.2
Do you think that sheltering is useful to reduce the dose of radiation exposure?		
Yes	199	17.0
Probably	513	43.8
Probably No	368	31.4
No	92	7.8
Do you know of a public shelter to evacuate to during a nuclear emergency?		
Yes	487	41.6
No	685	58.4
Did you know about SI before the study?		
Yes	716	61.1
No	456	38.9
Do you think that implementing SI prophylaxis can prevent exposure from all radionuclides?		
Yes	24	2.0
Probably	371	31.7
Probably No	617	52.6
No	160	13.7
Do you want to receive PDSI for your children?		
Yes	973	83.0
No	199	17.0
Do you want to implement SI prophylaxis for your children in a nuclear emergency?		
Yes	992	84.6
No	180	15.4
Do you think that pregnant women should also implement SI prophylaxis in a nuclear emergency?		
Yes	223	19.0
Probably	653	55.7
Probably No	234	20.0
No	62	5.3
Do you feel anxious about implementing SI prophylaxis for your children?		
Yes	261	22.3
Probably	580	49.5
Probably No	279	23.8
No	52	4.4

**Fig. 2:**
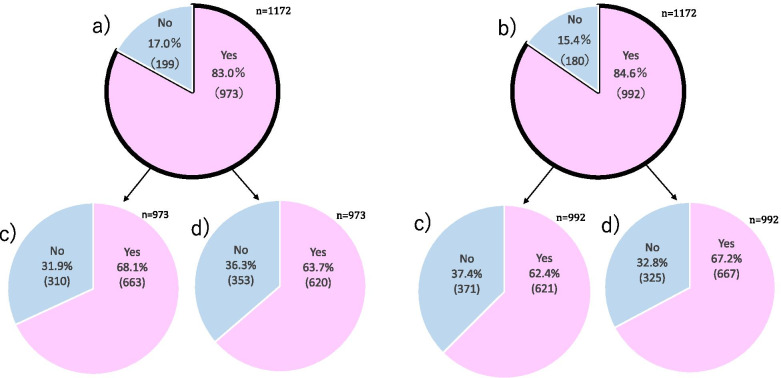
Awareness of SI among guardians living in the UPZ of the Genkai Nuclear Power Plant. **a** Do you want to receive PDSI for your children? **b** Do you want to implement SI prophylaxis for your children in a nuclear emergency? **c** Did you know about SI before the study? **d** Do you feel anxious about implementing SI prophylaxis for your children? Note. UPZ = Urgent Protective action planning Zone, SI = stable iodine

Of the 1172 guardians who responded to the questionnaire and were living in the UPZ, the 973 (83.0%) who wanted to receive PDSI and the 199 (17.0%) who did not want to receive PDSI were classified into Group 1 and Group 2, respectively. Of the 973 guardians in Group 1, 310 (31.9%) had not known about SI before the study, and 620 (63.7%) felt anxious about prophylactic SI for their children. In addition, of the 992 guardians who wished to receive prophylactic SI for their children, 371 (37.4%) had not known about SI before the study, and 667 (67.2%) felt anxious about prophylactic SI for their children during a nuclear emergency (Fig. 2).

Table 2 shows the comparison of guardians who wanted to receive PDSI (Group 1) and those who did not want to receive PDSI (Group 2). The guardians in Group 1 were significantly more interested in participating in a nuclear emergency drill (*n* = 658; 67.6%) compared with those in Group 2 (*n* = 90; 45.2%; *p* < 0.01) and were more interested in participating in a lecture about the health effects of radiation exposure (*n* = 593; 60.9%) compared with those in Group 2 (*n* = 83; 41.7%; *p* < 0.01). In addition, the guardians in Group 1 were significantly more likely to think that sheltering is useful to reduce the dose of radiation exposure (*n* = 611; 62.8%) than those in Group 2 (*n* = 101; 50.7%; *p* < 0.01). The guardians in Group 1 had known significantly more about SI before the study (*n* = 620, 63.7%) than those in Group 2 (*n* = 96; 48.2%; *p* < 0.01) and believed that implementing SI prophylaxis could prevent exposure from all radionuclides (*n* = 364; 37.4%) than those in Group 2 (*n* = 31; 15.6%; *p* < 0.01). The guardians in Group 1 also showed significantly greater desire to implement SI prophylaxis for their children in a nuclear emergency (*n* = 912; 93.7%) compared with those in Group 2 (*n* = 80; 40.2%; *p* < 0.01), and significantly more thought that pregnant women should also implement SI prophylaxis in a nuclear emergency (*n* = 804; 82.6%) compared with those in Group 2 (*n* = 72; 36.2%; *p* < 0.01). On the other hand, the guardians in Group 2 felt significantly more anxious about implementing SI prophylaxis for their children (*n* = 178; 89.4%) than those in Group 1 (*n* = 663; 68.1%; *p* < 0.01).

**Table 2:** Comparison of guardians who wanted to receive PDSI (Group 1) and those who did not want to receive PDSI (Group 2)

	Unit	Total *N* = 1172, n (%)	Group 1 *n* = 973, n (%)	Group 2 *n* = 199, n (%)	*P*
Age < 39 y	Yes	850 (72.5)	704 (72.4)	146 (73.4)	0.42
Have you participated in a nuclear emergency drill?	Yes	215 (18.3)	183 (18.8)	32 (16.1)	0.42
Do you want to participate in a nuclear emergency drill?	Yes	973 (83.0)	658 (67.6)	90 (45.2)	< 0.01
Do you want to participate in a lecture about the health effects of radiation exposure?	Yes	676 (57.7)	593 (60.9)	83 (41.7)	< 0.01
Do you think that sheltering is useful to reduce the dose of radiation exposure?	Yes	712 (60.8)	611 (62.8)	101 (50.7)	< 0.01
Did you know of a public shelter to evacuate to during a nuclear emergency?	Yes	487 (41.6)	407 (41.8)	80 (40.2)	0.37
Did you know about SI before the study?	Yes	716 (61.6)	620 (63.7)	96 (48.2)	< 0.01
Do you think that implementing SI prophylaxis can prevent exposure from all radionuclides?	Yes	395 (33.7)	364 (37.4)	31 (15.6)	< 0.01
Do you want to implement SI prophylaxis for your children in a nuclear emergency?	Yes	992 (84.6)	912 (93.7)	80 (40.2)	< 0.01
Do you think that pregnant women should also implement SI prophylaxis?	Yes	876 (74.7)	804 (82.6)	72 (36.2)	< 0.01
Do you feel anxious about implementing SI prophylaxis for your children?	Yes	841 (71.8)	663 (68.1)	178 (89.4)	< 0.01

Chi-squared tests

Group 1 = want to receive PDSI, Group 2 = not want to receive PDSI

*PDSI* Pre-distribution of stable iodine, *SNS* Social network services, *SI* Stable iodine

No significant differences were found between Groups 1 and 2 in the age of the guardians (< 39 years [72.4%] vs. ≥40 years [73.4%]; *p* = 0.42), participation in a nuclear emergency drill (18.8% vs. 16.1%; *p* = 0.42), and knowledge of a public shelter near their house to evacuate to during a nuclear emergency (41.8% vs. 40.2%; *p* = 0.37).

As shown in Table 3, the results of the binary logistic regression analysis showed that the following factors were independently associated with the desire to receive PDSI (reference, Group 1): thought that pregnant women should also implement SI prophylaxis in a nuclear emergency (odds ratio [OR] = 6.57, 95% confidence interval [CI]: 4.62–9.35; *p* < 0.01); want to participate in a lecture about the health effects of radiation exposure (OR = 1.99, 95%CI: 1.40–2.82; *p* < 0.01); thought that implementing SI prophylaxis can prevent exposure from all radionuclides (OR = 1.93, 95%CI: 1.24–2.99; *p* < 0.01); did know about SI before the study (OR = 1.91, 95%CI: 1.35–2.71; *p* < 0.01); and anxiety about implementing SI prophylaxis for their children (OR = 0.33, 95%CI: 0.20–0.55; *p* < 0.01).

**Table 3:** Results of the binary logistic regression analysis for guardians who wanted to receive PDSI. *N* = 1172

Variable	Unit	OR (95%CI)	*P*
Thought that pregnant women should also implement SI prophylaxis in a nuclear emergency	Yes/No	6.57 (4.62–9.35)	< 0.01
Want to participate in a lecture about the health effects of radiation exposure	Yes/No	1.99 (1.40–2.82)	< 0.01
Thought that implementing SI prophylaxis can prevent exposure from all radionuclides	Yes/No	1.93 (1.24–2.99)	< 0.01
Did know about SI before the study	Yes/No	1.91 (1.35–2.71)	< 0.01
Anxiety about implementing SI prophylaxis for their children	Yes/No	0.33 (0.20–0.55)	< 0.01

*OR* Odds ratio, *95%CI* 95% confidence interval, *PDSI* Pre-distribution of stable iodine, *SI* Stable iodine

## Discussion

The present study clarified the characteristics and awareness of protection against ionizing radiation, such as sheltering, SI, and listening to a lecture or participating in a nuclear emergency drill, of guardians living in a UPZ of a GNPP with an operating NPP in Japan.

In an NPP accident, it is first recommended that the residents living in the UPZ shelter in their house or a public shelter in accordance with the nuclear emergency response guideline from the NRA [6, 7]. Although only sheltering is not a perfect action to protect against ionizing radiation, sheltering is a relatively simple action to reduce internal and external radiation exposure from various radionuclides released into the environment, including radioactive iodine in the early phase of a nuclear accident [19]. However, it was found that approximately 40% of guardians thought that sheltering is not useful to reduce radiation exposure. The Japanese government reported the results of research about the effects of sheltering on human bodies; compared to staying outdoors, sheltering in a standard wooden house can reduce external radiation exposure by about 60% in Japanese persons. In addition, a standard rebar house can reduce external radiation exposure by about 90% compared to staying outdoors. Furthermore, internal exposure can be reduced by about 30% compared to staying outdoors, regardless of the type of house materials without ventilation [20].

On the other hand, the present study found that approximately 60% of guardians did not know of a public shelter to evacuate to during a nuclear accident. The recommendation of the World Health Organization (WHO) for a nuclear emergency is that, in areas further away from an NPP emergency, PDSI to households is not considered feasible, and stocks of SI should be stored strategically at, for example, schools, hospitals, pharmacies, fire stations, police stations, and civil defense centers such as public shelters [13]. Therefore, when a nuclear accident occurs, if the residents living in the UPZ do not have their own SI, they receive the SI and implement SI prophylaxis in the public shelter and then evacuate to outside of the UPZ area [20]. These results regarding their awareness about sheltering suggest that a strong message about the effectiveness of sheltering in their houses or public shelters and more information about where local public shelters need to be provided to the residents living in a UPZ before a nuclear accident.

The present study also found that 83.0% of guardians wanted to receive PDSI, and 84.6% of the guardians wanted to implement SI prophylaxis for their children in a nuclear emergency. However, among the guardians who wanted to receive PDSI, 31.9% had not known about SI before the study, and 63.7% felt anxious about prophylactic SI for their children. Similarly, of the guardians who wanted to implement SI prophylaxis for their children in a nuclear emergency, 37.4% had not known about SI before the study, and 67.2% felt anxious about prophylactic SI for their children. These results show conflict between the guardian’s awareness of SI, in other words, they wanted to receive PDSI and implement SI prophylaxis for their children in a nuclear emergency, but many of them felt anxious about implementing SI prophylaxis for their children. The groups most likely to benefit from implementing SI prophylaxis are children, adolescents, and pregnant and breastfeeding women [10, 21], so priority should be given to children and younger adults. However, pregnant women and guardians parenting young children tend to be cautious about the side effects of drugs in general, not only implementing SI prophylaxis [6]. Side effects of implementing SI prophylaxis are rare and include iodine-induced transient hyperthyroidism or hypothyroidism and allergic reactions [21, 22]. For these reasons, it is important to communicate with pregnant women and guardians of young children about the risks associated with implementing SI prophylaxis for their children, including the benefits and side effects. Points to consider in the present study were that 33.7% of the guardians thought that SI could prevent exposure from all radionuclides. Implementing SI prophylaxis can block or reduce the accumulation only of radioactive iodine in the thyroid [19]. Implementing SI prophylaxis within 1 to 2 h before inhalation of and exposure to I-131 can block > 90% of thyroid uptake of I-131 [23] and implementing SI prophylaxis is most effective in the first few hours of internal exposure. However, in the case of a nuclear emergency, stand-alone protective action, such as only prophylactic SI, should not be considered [19]. Nevertheless, there is a popular misconception that SI is a silver bullet that protects against harm from radiation exposure [13]. Even in such a difficult situation, education is the key to disaster prevention, preparation, response, and recovery [24]. Approximately half of the guardians wanted to participate in a nuclear emergency drill and lecture about the health effects of radiation exposure, whereas only 18.3% of respondents had participated in a nuclear emergency drill. Moreover, it was found that those who wanted PDSI, compared to those who did not want PDSI, were about twice as willing to participate in a lecture about the health effects of radiation exposure. All local municipalities in which NPPs are located have a responsibility to perform a nuclear emergency drill and hold a lecture about protection against ionizing radiation [6], even though, because of the low number of health communication professionals, opportunities to participate in such drills and lectures are lacking even for UPZ residents [25, 26]. As for future perspectives, it is important for professionals belonging to radiation research institutes or universities and local municipalities located near NPPs to work together to educate residents living in a UPZ. Furthermore, it is essential to train specialists to work in a UPZ and improve risk communication about protection against ionizing radiation, including implementing SI prophylaxis, to raise guardian awareness in preparation for a nuclear accident.

There are several limitations of the present study. First, since the study analyzed only one area of a UPZ of an operating NPP in Japan, there might be sampling bias. Second, it was not possible to accurately determine the number of questionnaires distributed because teachers at each kindergarten were requested to distribute them. Some kindergartens did not provide the number of questionnaires passed on to the guardians.

Third, due to further potential sampling bias, such as respondents living mainly in a UPZ, differences depending on area of residence, such as a PAZ or over 30 km, cannot be analyzed. Awareness of the need for protection against ionizing radiation might vary depending on the distance from an NPP. Fourth, demographic factors other than sex, age, and number of children under 18 years of age were not collected. Knowledge of implementing SI prophylaxis might have a relationship to academic background, occupation, or place of birth. Finally, an explanation about the implementation of SI prophylaxis for guardians who did not know about SI was provided before the questionnaire; though the content of the questionnaire was not explained, it may have had some effect on the answers.

Although there are several limitations, the present study provides effective baseline data on the awareness of the need for protection against ionizing radiation of restarted NPPs among those living in a UPZ a decade after the FDNPP accident.

## Data Availability

The datasets used and analyzed during the current study are available from the corresponding author on reasonable request.
